# The Effect of the Thermosensitive Biodegradable PLGA–PEG–PLGA Copolymer on the Rheological, Structural and Mechanical Properties of Thixotropic Self-Hardening Tricalcium Phosphate Cement

**DOI:** 10.3390/ijms20020391

**Published:** 2019-01-17

**Authors:** Lucy Vojtova, Lenka Michlovska, Kristyna Valova, Marek Zboncak, Martin Trunec, Klara Castkova, Milan Krticka, Veronika Pavlinakova, Petr Polacek, Matej Dzurov, Vera Lukasova, Michala Rampichova, Tomas Suchy, Radek Sedlacek, Maria-Pau Ginebra, Edgar B. Montufar

**Affiliations:** 1 CEITEC – Brno University of Technology, Purkynova 656/123, 612 00 Brno, Czech Republic; lenka.michlovska@ceitec.vutbr.cz (L.M.); kristyna.valova@ceitec.vutbr.cz (K.V.); marek.zboncak@ceitec.vutbr.cz (M.Z.); martin.trunec@ceitec.vutbr.cz (M.T.); klara.castkova@ceitec.vutbr.cz (K.C.); veronika.svachova@ceitec.vutbr.cz (V.P.); petr.polacek@ceitec.vutbr.cz (P.P.); xcdrzurov@fch.vut.cz (M.D.); 2 Trauma Surgery Department, The University Hospital Brno, Jihlavska 340/20, 325 00 Brno, Czech Republic; mil.krticka@gmail.com; 3 Lab of Tissue Engineering, Institute of Experimental Medicine, Czech Academy of Sciences, Videnska 1083, 14240 Prague, Czech Republic; vera.lukasova@iem.cas.cz (V.L.); michala.rampichova@iem.cas.cz (M.R.); 4 Department of Composites and Carbon Materials, Institute of Rock Structure and Mechanics, The Czech Academy of Sciences, V Holesovickach 41, 182 09 Prague, Czech Republic; suchy@irsm.cas.cz; 5 Department of Mechanics, Biomechanics and Mechatronics, Faculty of Mechanical Engineering, Czech Technical University in Prague, Technicka 4, 166 07 Prague, Czech Republic; radek.sedlacek@fs.cvut.cz; 6 Biomaterials, Biomechanics and Tissue Engineering Group, Department of Materials Science and Metallurgical Engineering, Universitat Politècnica de Catalunya, 08019 Barcelona, Spain; maria.pau.ginebra@upc.edu

**Keywords:** injectable bone cements, thixotropic, rheology, morphology, kinetics, biocompatibility

## Abstract

The current limitations of calcium phosphate cements (CPCs) used in the field of bone regeneration consist of their brittleness, low injectability, disintegration in body fluids and low biodegradability. Moreover, no method is currently available to measure the setting time of CPCs in correlation with the evolution of the setting reaction. The study proposes that it is possible to improve and tune the properties of CPCs via the addition of a thermosensitive, biodegradable, thixotropic copolymer based on poly(lactic acid), poly(glycolic acid) and poly(ethylene glycol) (PLGA–PEG–PLGA) which undergoes gelation under physiological conditions. The setting times of alpha-tricalcium phosphate (α-TCP) mixed with aqueous solutions of PLGA–PEG–PLGA determined by means of time-sweep curves revealed a lag phase during the dissolution of the α-TCP particles. The magnitude of the storage modulus at lag phase depends on the liquid to powder ratio, the copolymer concentration and temperature. A sharp increase in the storage modulus was observed at the time of the precipitation of calcium deficient hydroxyapatite (CDHA) crystals, representing the loss of paste workability. The PLGA–PEG–PLGA copolymer demonstrates the desired pseudoplastic rheological behaviour with a small decrease in shear stress and the rapid recovery of the viscous state once the shear is removed, thus preventing CPC phase separation and providing good cohesion. Preliminary cytocompatibility tests performed on human mesenchymal stem cells proved the suitability of the novel copolymer/α-TCP for the purposes of mini-invasive surgery.

## 1. Introduction

Calcium phosphate bone cements (CPCs) have been of interest in the field of bone regeneration for over 30 years. CPCs are hydraulic cements composed of calcium orthophosphate which forms a mouldable paste following mixing with a liquid phase generally consisting of pure water or aqueous sodium phosphate, physiological saline or phosphate buffered saline [1]. CPCs are also tagged as self-setting materials due to their ability to harden in vivo via a chemical setting reaction under physiological conditions [2,3]. One of the main positive aspects of CPCs consists of their injectability which allows for the cement to be implanted by means of mini-invasive surgical procedures. Thus, they have significant potential with respect to the fields of orthopaedics, traumatology (fracture fixation) and dentistry. However, commercial CPCs do not yet feature all the desired properties; in particular, they are not completely injectable at low force levels and with high solid contents without the occurrence of separation, and demonstrate washout resistance to body fluids soon after mixing. Supplementing CPCs with polymeric additives in different forms appears to provide a promising strategy for the avoidance of the afore-mentioned limitations. A number of polymers have been explored with respect to improving the properties of CPCs, such as natural hydrogels based on collagen [4,5], gelatine [6], carboxymethylcellulose [7], hyaluronic acid [7], a hyaluronan-bisphosphonate composite [7], chitosan [8], synthetic water-soluble poly (ethylene glycol) [9] and biodegradable poly(propylene fumarate) crosslinked with hydroxyethyl methacrylate [10]. Moreover, a further, novel CPC design involving a thermo-responsive copolymer known as Poloxamer has recently been demonstrated [11]. Poloxamer, a triblock copolymer of poly (ethylene glycol-*b*-propylene glycol-*b*-ethylene glycol) [12], thermogels at body temperature, thus increasing the viscosity of the CPC immediately following injection into the site of the defect and enhancing washout resistance [11]. However, Poloxamer is not biodegradable, it is soluble only in physiological fluids and exhibits toxicity at higher concentrations [12]. 

This study investigates a biodegradable, non-toxic, thermosensitive copolymer based on FDA-approved poly(lactic acid), poly(glycolic acid) and poly(ethylene glycol) (PLGA–PEG–PLGA), commercially known as the ReGel^®^ or OncoGel^®^ drug delivery systems incorporating insulin and the drug Paclitaxel, respectively. In a similar way to Poloxamer, the PLGA–PEG–PLGA copolymer forms a free-flowing sol at room temperature and a physical three-dimensional network hydrogel at body temperature resulting in very good injectability due to its thixotropic rheological behaviour [13]. Moreover, PLGA–PEG–PLGA is fully biodegradable in the Krebs cycle to carbon dioxide and water [14]. However, the use of PLGA–PEG–PLGA as a drug delivery system is limited, since the copolymer exhibits a low degree of functionality; hence, a great deal of attention has been devoted to PLGA–PEG–PLGA functionalization [15,16]. Recently, we modified the PLGA–PEG–PLGA copolymer via the addition of inorganic bioactive hydroxyapatite (HA) in the form of micro-, nano- and core-shell particles [17] and the results revealed that these composites inherited the sol-gel and gel-sol phase transitions of the polymer solutions. These characteristics subsequently led to the assumption that the addition of the PLGA–PEG–PLGA copolymer may provide for the formation of a new class of CPCs with properties closer to the ideal. 

The aim of this study, therefore, was to assess the effect of a thermo-sensitive PLGA–PEG–PLGA copolymer on the setting reaction, injectability and cohesive properties of a CPC that sets due to the hydrolytic transformation of alpha-tricalcium phosphate (α-TCP) into calcium deficient hydroxyapatite (CDHA) according to the following chemical reaction (1).

(1) $$3\;\alpha -Ca_{3}{\left(PO_{4}\right)}_{2}+H_{2}O\;\to Ca_{9}\left(HPO_{4}\right){\left(PO_{4}\right)}_{5}OH$$

Emphasis was placed on correlating the time-dependent rheological behaviour of the cement with the kinetics of the setting reaction, injectability and washout resistance. Finally, the degradation behaviour and cytocompatibility of the cement were assessed in views of its potential application for bone regeneration purposes.

## 2. Results

### 2.1. Synthesis and Characterization of the PLGA–PEG–PLGA Copolymer

#### 2.1.1. Copolymer Composition

A well-defined “smart” poly(lactic acid-*co*-glycolic acid)-*b*-poly(ethylene glycol)-*b*-poly(lactic acid-*co*-glycolic acid) (PLGA–PEG–PLGA) triblock copolymer with a PLGA/PEG weight ratio equal to 2.47 and a PLA/PGA molar ratio equal to 2.96 was synthesized via ring opening polymerization. The number-average molecular weight *M_n_* and the molar composition of the samples were determined using ^1^H NMR spectroscopy and calculated via a comparison of lactic acid methylene (1.55 and 5.17 ppm), glycolic acid methylene (4.80 ppm) and PEG methylene (3.65 ppm) integrations as we reported previously [18] (Supplementary Figure S1). The molecular weight determined by means of ^1^H NMR (5210 g·mol^−1^) and GPC (5300 g·mol^−1^) exhibited very good agreement with the theoretical value (5250 g·mol^−1^). The copolymer had a narrow molecular weight distribution with an *M_w_*/*M_n_* ratio equal to 1.23 (obtained via GPC, see Supplementary Figure S2).

#### 2.1.2. Sol-Gel Transitions

**Figure 1:**
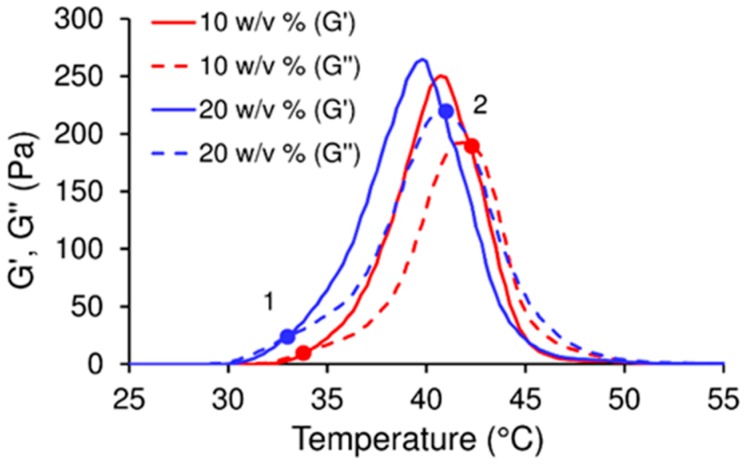
The visco-elastic properties of the 10 *w*/*v*% and 20 *w*/*v*% poly(lactic acid), poly(glycolic acid) and poly(ethylene glycol) (PLGA–PEG–PLGA) aqueous solutions (red and blue lines respectively). “1” represents the sol-gel transition (start of gelation) and “2” the gel-sol transition (end of gelation).

The rheological analysis was employed in order to confirm the assumptions concerning the thermo-sensitivity and flow properties of the PLGA–PEG–PLGA amphiphilic copolymer. Due to hydrophobic interactions, PLGA chains have the ability to form “flower-like” micelles in water with increasing temperature. This leads to gelation of the copolymer at the physiological temperature (37 °C), followed by phase-separation and micelles dehydration at a higher temperature (above 45 °C). Hence, two phase transitions, sol-to-gel and gel-to-sol occur. In the case of rheological analysis, the first transition (sol-gel) is defined by means of the gelation temperature as the intersection of the storage modulus (G′) and the loss modulus (G″) when G′ becomes greater than G″. The second G′ and G″ intersection is caused by a rapid decline in G′ to below G″, at which point the gel structure starts to collapse which, in turn, is followed by the loss of the visco-elastic properties of the material. The rheological behaviour of both the 10 *w*/*v*% and 20 *w*/*v*% PLGA–PEG–PLGA aqueous solutions were evaluated within a temperature range of between 15 and 60 °C (Figure 1). Figure 1 shows that the first intersection (sol-gel transition) of the two solutions progresses at 33.7 °C and the second (end of gelation) occurs at 42.5 °C, thus providing evidence of the thermo-sensitivity of both the 10 *w*/*v*% and 20 *w*/*v*% PLGA–PEG–PLGA solutions and their ability to gel under physiological conditions. 

### 2.2. Alpha-Tricalcium Phosphate Preparation and Characterization

α-TCP powder (Supplementary Figure S3a) with a narrow monomodal particle size distribution, a mean size of 3.1 μm (Supplementary Figure S3b) and specific surface area of 2.4 m^2^·g^−1^ was synthetized at 1400 °C for 2 h and milled according to previously verified published protocols [19]. The 100% purity of the α-TCP powder was confirmed by means of wide-angle X-ray scattering (WAXS) analysis (see Figure 5a—the lowest curve).

### 2.3. Rheology and Self-Setting Reaction of the Copolymer/α-TCP Cements

**Figure 2:**
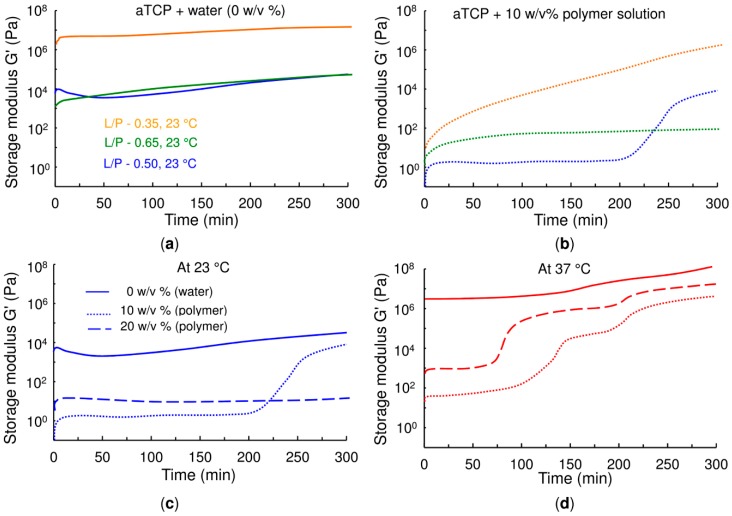
Time-sweep curves of alpha-tricalcium phosphate (α-TCP) cement pastes with (**a**) and (**b**) differing liquid to powder (L/P) ratios (0.35; 0.5; 0.65 g·g^−1^) and liquid phases (0 *w*/*v*% water and 10 *w*/*v*% polymer solution) at 23 °C (the curves determined for the α-TCP cement pastes with the 20 *w*/*v*% polymer solution are shown in Supplementary Figure S4), (**c**) and (**d**) a constant L/P ratio of 0.5 g·g^−1^ and differing polymer solution concentrations (0 *w*/*v*%, 10 *w*/*v*% and 20 *w*/*v*% polymer solutions) at 23 and 37 °C, respectively.

The influence of copolymer concentration and temperature on the self-hardening process of the modified CPCs was evaluated by means of rheological measurement. The CPC samples prepared from both 10 *w*/*v* % and 20 *w*/*v*% polymer solutions as the liquid phases were compared to a CPC of *α*-TCP and pure water (0 *w*/*v*%) with no other additives at both 23 °C and 37 °C. Figure 2 and Supplementary Figure S4 provide evaluations of the CPC shear elastic modulus (G′) over a time sweep of 5 h in a linear frequency regime (1 Hz) and applying a low strain value (0.5%). Figure 2a,b and Supplementary Figure S4 show the measurement of the CPC shear elastic modulus at 23 °C for differing water/*α*-TCP and copolymer/*α*-TCP liquid to powder (L/P) ratios, whereas Figure 2c,d compare the influence of temperature (23 and 37 °C) on the setting of the cement with respect to differing polymer concentrations.

A comparison of Figure 2a,b reveals that after 1 min of mixing involving the placing of pastes in a rheometer set-up, the water/α-TCP cements, which commenced rheological measurement at a G′ of higher than 1 kPa and more than 1 MPa for an L/P equal to 0.35 g·g^−1^, were stiffer than the copolymer/α-TCP cements. In terms of injectability through a cannula with an inner diameter of 2 mm, it was found to be difficult to inject the 0.5 and 0.65 g·g^−1^ L/P ratios, whereas it was impossible to inject the cement with an L/P equal to 0.35 g·g^−1^ due to its high level of stiffness. In contrast, those copolymer/α-TCP cements with a G′ of below 1 kPa were found to be totally injectable soon after mixing. It is clear from the blue line in Figure 2b corresponding to an L/P equal to 0.5 g·g^−1^ that the storage modulus remains low and almost constant up to 200 min. This period is known as the lag time (or lag phase) which, as will be shown later, corresponds principally to the dissolution of α-TCP which leads to the nucleation of CDHA crystals as described in Equation (1). Once the nucleation of the CDHA crystals commences, fostered by the local saturation of Ca^2+^ and PO_4_^2-^ ions, originating from the dissolution of α-TCP, the storage modulus of the cement begins to increase progressively. Therefore, the time at which the modulus begins to increase can be considered to represent the initial setting time of the cement.

When the L/P ratio was decreased to 0.35 g·g^−1^, as shown in Figure 2b, the storage modulus progressively increased from as early as at the beginning of the test, i.e., this cement formulation did not exhibit a lag time. The reason for this phenomenon is that the lower L/P ratio implies the better packaging of the *α*-TCP particles due to the smaller inter-particle spaces and, therefore, a lower volume of liquid is translated into the more rapid saturation of ions for the nucleation of the CDHA. In contrast, when the L/P ratio was increased to 0.65 g·g^−1^, the volume of liquid was so high that no increment in the storage modulus was observed during the experiment; hence the setting time of this formulation was longer than 300 min. 

With respect to the water/α-TCP cements, despite the tendency of the storage modulus to increase, no clear sharp increase in stiffness up to 300 min was observed, which suggests that the first stage of the setting reaction of α-TCP mixed with water is slower than that following its mixing with a copolymer; this was probably due to the higher degree of acidity (pH of 2.8) of the copolymer solution than that of pure water (pH of 6.7) which fostered the dissolution of the α-TCP.

The effect of temperature and copolymer concentration on the storage modulus can be observed in Figure 2c,d. Similarly to the increase in the L/P ratio, an increment in the copolymer concentration at 23 °C from 10 *w*/*v*% to 20 *w*/*v*% was found to increase the lag time (to more than 300 min in the case of the 20 *w*/*v*% of the copolymer). In addition, it can be observed that the storage modulus within the lag time was also higher concerning the 20 *w*/*v*% concentration, the reason for which is that the higher the concentration of the polymer, the more compact is the micellar structure, thus resulting in the increased stiffness of the cement (Figure 1). Increasing the temperature to a biological 37 °C increase the storage modulus within the lag time due to the commencement of the gelation of the PLGA–PEG–PLGA at 33 °C, as shown in Figure 1. In addition, a reduction in the lag time was observed due to the more rapid rate of *α*-TCP dissolution at higher temperatures. Conversely, when water rather than a copolymer was used in the preparation of the CPC, the initial storage modulus was so high at 37 °C (more than 5 MPa) that it inhibited the injection of the cement and even the moulding thereof with a spatula.

### 2.4. Visco-Elastic Properties of Copolymer/α-TCP Cements

**Figure 3:**
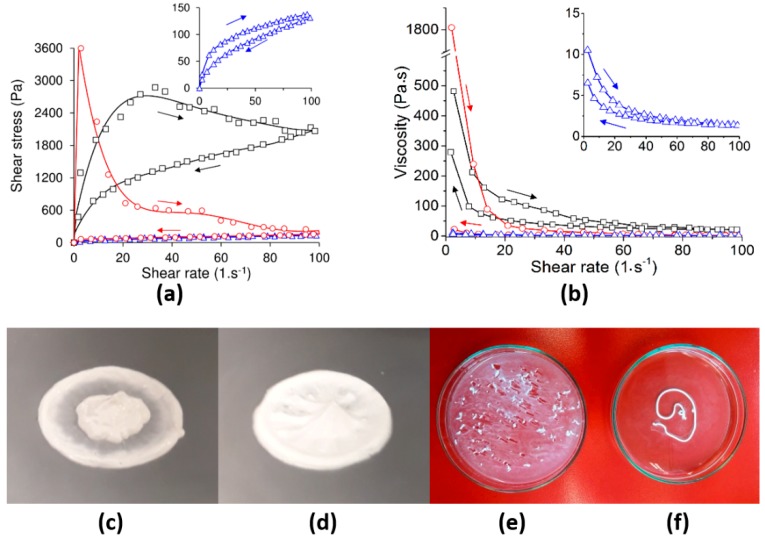
Steady rheological analysis and the viscosity curves of the copolymer solution and CPCs after 1 min of setting. Relationship between shear stress and the shear rate (**a**) and relationship between viscosity and the shear rate (**b**) of the 20 *w*/*v*% copolymer solution (blue line with triangles), α-TCP paste with deionized water (red line with circles) and α-TCP paste with 20 *w*/*v*% copolymer solution (black line with squares), prepared at an L/P of 0.5 g·g^−1^ at 25 °C. The arrows indicate the outward and return sweeps; the hysteresis loop indicates thixotropy. While the α-TCP cement made from the addition of pure water separated following steady state rheological measurement (**c**) and disintegrated following immediate injection in water at 37 °C (**e**), the α-TCP cement made from the addition of the 20 *w*/*v*% copolymer solution remained homogeneous (**d**) and exhibited cohesion following injection in water at 37 °C, retaining the shape of the injected filament (**f**).

The steady rheological analysis of the CPCs within the first 40 s following the mixing of the liquid and powder phases was conducted so as to ascertain the influence of the PLGA–PEG–PLGA thermo-sensitive triblock copolymer on the initial stiffness of the pastes, the parameter that determines both the degree of injectability and cohesion properties thereof. Figure 3a,b show the results of the dependence of shear stress and viscosity respectively on the shear rate for a pure copolymer solution (blue triangles), CPC with pure water (red circles) and CPC with a 20 *w*/*v*% copolymer solution (black squares). 

The pure copolymer solution exhibited shear thinning behaviour, i.e., an asymptotic increase in the shear stress and an exponential decrease in viscosity with the shear rate. The copolymer exhibited the rapid recovery of the viscous state following a decrease in the shear rate (small hysteresis loop), thus suggesting pseudoplastic rather than thixotropic rheological behaviour. The two *α*-TCP pastes also exhibited shear thinning rheological behaviour, but at different orders of magnitude of both shear stress and viscosity. The cements also exhibited a Bingham yield stress of around 1200 Pa when the *α*-TCP was mixed with the copolymer and as high as 3600 Pa when mixed with water. A further important difference between the two CPCs and the copolymer alone consisted of the occurrence of larger hysteresis loops (the loop area of the water/*α*-TCP was 65,117 Pa·s^−1^ and the loop area of the copolymer/*α*-TCP was 81,150 Pa·s^−1^) than those of the pure polymer (loop area of 1820 Pa·s^−1^), thus revealing the higher degree of cement thixotropy. Even though both cements were thixotropic, significant differences were evident in terms of their behaviour. The *α*-TCP mixed with pure water exhibited a sharp increase in shear stress followed by a drastic stress decrease after reaching the maximum, attaining a value close to 200 Pa at the maximum shear rate. In addition, no recovery of shear stress and viscosity was observed following the decrease in the shear rate. In contrast, the copolymer/*α*-TCP paste displayed a progressive increase in shear stress with the shear rate. The shear stress decreased slowly with the shear rate after reaching the maximum point, but maintained a value of around 2000 Pa at the maximum shear rate. Once the shear rate was reduced, the shear stress continued to decrease, accompanied by a partial recovery in viscosity.

Figure 3c–f display representative images of the cement pastes following rheological testing and injection in water at 37 °C. It can be observed that the water/*α*-TCP paste separated during the rheological test and disintegrated in contact with water (Figure 3c,e). In contrast, the copolymer/CPC paste remained homogeneous during the rheological test and retained the shape of the injected filament in contact with water, thus displaying good cohesive behaviour (Figure 3d,f).

Table 1 provides a summary of the physical parameters of the copolymer/α-TCP (pH and complex viscosities) together with its injectable/cohesion properties based on the L/P ratio and the amount of copolymer (0; 10 and 20 *w*/*v*%) within the lag phase, specifically 3 min following the mixing of the copolymer and α-TCP. It is clear that the CPCs with water as the liquid phase (0 *w*/*v*% of the copolymer) exhibit a basic pH of around 8.5 with concern to all the L/P ratios and very high complex viscosity levels that decrease gradually with an increase in the L/P ratio. Both of the cements that incorporated the copolymer (pH = 3) exhibit a pH value of around the physiological level (7.0–7.4) and low complex viscosities of between 0.9 and 43.4 Pa·s with respect to all the L/P ratios evaluated. Therefore, the more concentrated and more acidic is the copolymer solution, the faster is the setting process, as can be observed from the results of rheological measurement at 37 °C (Figure 2d). The reason is the more rapid dissolution of the α-TCP under acidic conditions [20] promoted by increases in temperature. The data proves that cohesion not only depends on the viscosity of the paste, but also on the thermo-sensitive properties of the copolymer in dependence with temperature.

### 2.5. The Effect of the Self-Setting Reaction on pH, Mass Loss and Crystalline Composition 

**Figure 4:**
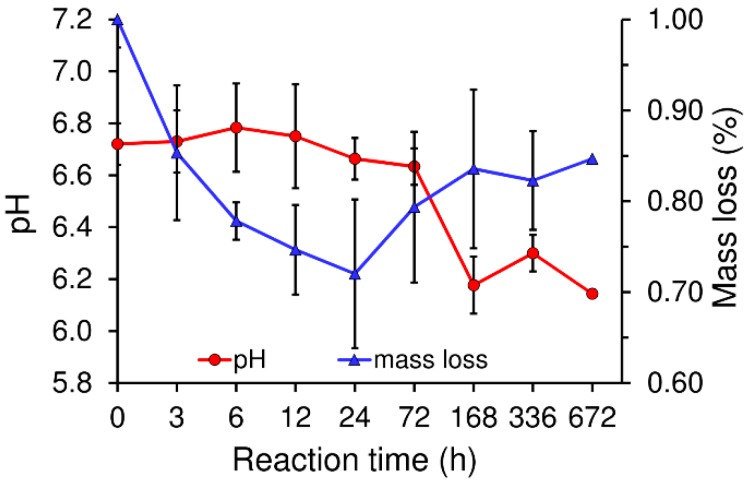
Change in the pH and mass loss of the 20 *w*/*v*% copolymer/*α*-TCP cement with an L/P of 0.5 g·g^−1^ at 37 °C and immersed in ultrapure water.

The mechanism of the initial setting of the cement can be studied by measuring the CPC mass loss and pH in the immersion liquid (water) in which the cement set. As can be observed in Figure 4, the copolymer/*α*-TCP mass decreased with time during the lag phase (*α*-TCP dissolution) up to 24 h accompanied by a slight increase in pH which reached a maximum after 6 h of reaction, reflecting the ionic concentration of calcium ions (Ca^2+^) which increased in the immersion medium due to the dissolution of *α*-TCP particles. When the mass loss increased, the pH trend was reversed, i.e., the Ca^2+^ was consumed due to the nucleation and initial growth of the CDHA crystals.

**Figure 5:**
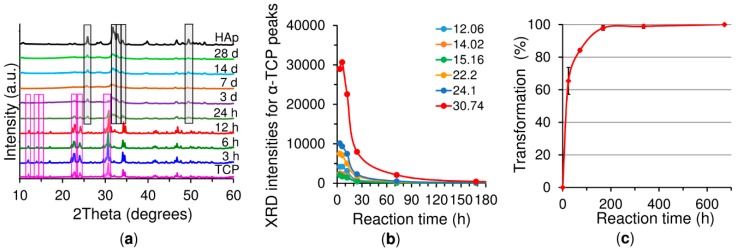
Crystalline phase analysis of the 20 *w*/*v*% polymer/CPC over a reaction time of 3 h to 28 days at a fixed L/P ratio of 0.5 g·g^−1^ at 37 °C. (**a**) Representative XRD patterns, apatite was detected as soon as after 24 h (grey plotted part) and α-TCP was detected up to the third day of reaction (purple plotted part). (**b**) Intensity of the XRD peaks at 12.06; 14.02; 15.16; 22.20; 24.10 and 30.74 °(2ϴ). (**c**) The total average transformation of α-TCP to calcium deficient hydroxyapatite (CDHA) while neglecting negative values at the 3, 6 and 12 h setting times.

The chemical reaction of the dissolution of the α-TCP particles and the precipitation of the CDHA crystals (reaction 1) can also be monitored by means of X-ray crystalline phase analysis (Figure 5a). Even though no CDHA formation signals at the 25.9, 31.75, 32.12, 32.78 and 49.5 °(2ϴ) diffraction reflections can be observed at reaction times below 6 h, it is evident that the intensity of the diffraction peaks of the *α*-TCP increases up to 6 h of the reaction, indicating that the most reactive part of the *α*-TCP dissolves at such short reaction times. For reasons of clarification, the intensity changes of the six diffraction peaks representing pure monoclinic crystalline α-TCP at 12.06; 14.02; 15.16; 22.20; 24.10 and 30.74 °(2ϴ) were compared at different reaction times (Figure 5b). It is evident that all the α-TCP peaks increase up to 6 h of reaction while the α-TCP peak intensities tend to decrease after 6 h, whereas the CDHA peaks appear after 12 h and increase in intensity in dependence with the reaction time, which represents the transformation of crystalline *α*-TCP to CDHA. All the *α*-TCP peaks totally disappeared between days 7 and 14 characterized by the 97.9% and 100% transformation of the *α*-TCP to CDHA respectively, without the occurrence of further inorganic phases [21] (Figure 5c). The presence of both α-TCP and CDHA patterns confirms that the dissolution-precipitation process takes place in parallel.

### 2.6. Microstructure and Microporosity

**Figure 6:**
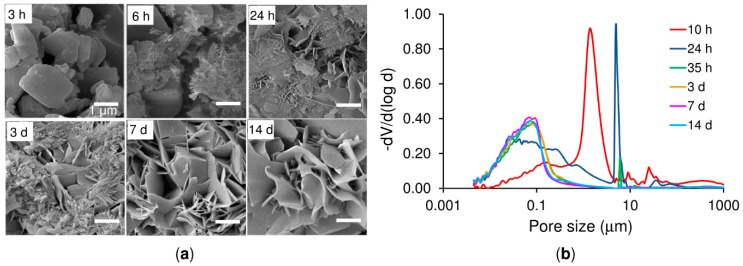
Microstructure and porosity development of the cement samples prepared at an L/P ratio of 0.5 g·g^−1^, *α*-TCP mixed with 20 *w*/*v*% of the copolymer at different reaction times at 37 °C. (**a**) Representative SEM images of the fracture surface for *α*-TCP pastes after 3 h, 6 h, 24 h, 3 d, 7 d and 14 d of reaction. The scale bar is 1 µm in all cases. (**b**) Pore size distribution during a reaction time from 10 h to 14 d.

The microstructures of cross-sections of 20 *w*/*v*% polymer/*α*-TCP during setting (L/P equal to 0.5 g·g^−1^), shown in Figure 6a, expose the morphology transformation of *α*-TCP particles during setting. The original particles can be seen to be smooth, polyhedral and of various sizes (3 h). After 6 h of reaction at 37 °C, however, the *α*-TCP surface had become surrounded by tiny precipitated CDHA crystals that grew in a radiating pattern from the original *α*-TCP particles as the reaction proceeded.

After 24 h of reaction, the CDHA crystals exhibited two distinguishable morphologies: Needle-like and plate-like crystals. In addition, a number of areas were observed with no crystals. The needle-like crystals were attributed to the rapid hydrolysis of the fine *α*-TCP powder and the plate-like crystals to the slower reaction kinetics of the coarse *α*-TCP powder [2]. The fact that both needle-like and plate-like crystals were observed with respect to a powder with such a narrow particle size distribution provides evidence that the hydrolysis process was ongoing. At day 3, the precipitated CDHA crystals were well-developed with sizes of micrometres in length and hundreds of nanometres in width. The CDHA crystals grew into each other resulting in a crystal-entangled cement microstructure. The microstructure remained almost unchanged from the third day onwards. The cement when set was highly porous (52 ± 1%) due to the free spaces between the CDHA crystals (7 d and 14 d) [2,22].

The pore size distribution was found to match with *α*-TCP hydrolysis mechanisms. The copolymer/*α*-TCP initially exhibited a bimodal pore size distribution with mainly large pores that progressively became transformed into a monomodal distribution of small pores (Figure 6b). Espanol et al. [22] attributed the larger pores (˃ 0.1 μm) to voids between CDHA crystal aggregates formed in the position of α-TCP particles, and the smaller pores of ˂ 0.1 μm to voids between the needle- and/or plate-like CDHA crystals that formed aggregates in the completely set cements. With respect to this study, porosity was seen to change along with the progress of the hydrolysis reaction. After 10 h of reaction, the main sharp peak in the pore size distribution appeared at around 2 μm, which corresponded to the space between the α-TCP particles. The second broad and smaller peak, between 0.5 and 0.01 μm, corresponded to the spaces between recently formed and still growing CDHA crystals. After 24 h of reaction, the size of the pores between the α-TCP particles increased to 8 μm due to the dissolution of the α-TCP particles resulting in an increase in the surface-to-surface distance between neighbouring particles. In parallel, the amount of inter-crystal voids increased, and their size distribution shifted to smaller values as a consequence of the progression of CDHA precipitation. At the 35 h time-point the amount of pores between the α-TCP particles, now consisting of CDHA aggregates, significantly decreased and their size shifted slightly to larger values. At the same time, the amount of inter-crystal voids continued to increase and followed the shift to smaller pore sizes (0.01–0.1 μm). No change was observed in the pore size distribution from the third day onwards. The final distribution was monomodal with no large pores and inter-crystal pores centred at small sizes below 0.1 μm. Those pores larger than 10 μm consisted of air bubbles introduced during the mixing of the paste. Furthermore, the changes in the microstructure that occurred during the reaction did not lead to a change in the total porosity of the cement (52 ± 1%).

### 2.7. Mechanical Testing and Degradation Study

**Figure 7:**
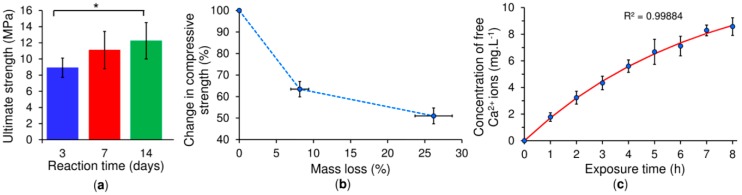
Results of the compression and accelerated degradation tests conducted on the 20 *w*/*v*% polymer/CPC at an L/P ratio of 0.5 g·g^−1^. Ultimate compressive strength during the reaction time of 3 to 14 days at 37 °C (**a**), * denotes statistically significant differences (*p* > 0.05) between the 14 day group and the 3 day group. Changes in the compressive strength of the same cement set for 10 days as a function of the mass loss; the points correspond to 0, 4 and 8 h of exposure to acidic conditions at 37 °C (**b**). The concentration of free Ca^2+^ ions in the supernatants following the exposure of the samples to acidic conditions (**c**); the red line highlights the fit of the experimental points with the first order release model according to $C=C_{\infty }-C_{0}\;exp{\left(-\lambda t\right)}^{\;}$. The calculated half-life time *λ* of the release of the Ca^2+^ ions was 4.68 h.

The results of the mechanical compression test performed on the CPC samples with the 20 *w*/*v*% copolymer and an L/P of 0.5 g·g^−1^ at days 3, 7 and 14 are depicted in Figure 7a. The compressive strength of the cement increased progressively with the reaction time, reaching a maximum after 14 days of reaction, at which time all the α-TCP had been converted to CDHA. The compressive strength of trabecular bone (0.6–15 MPa) [23] was in good agreement with the maximum strength of the copolymer/CPC on both day 7 (11.2 MPa) and day 14 (12.2 MPa).

**Figure 8:**
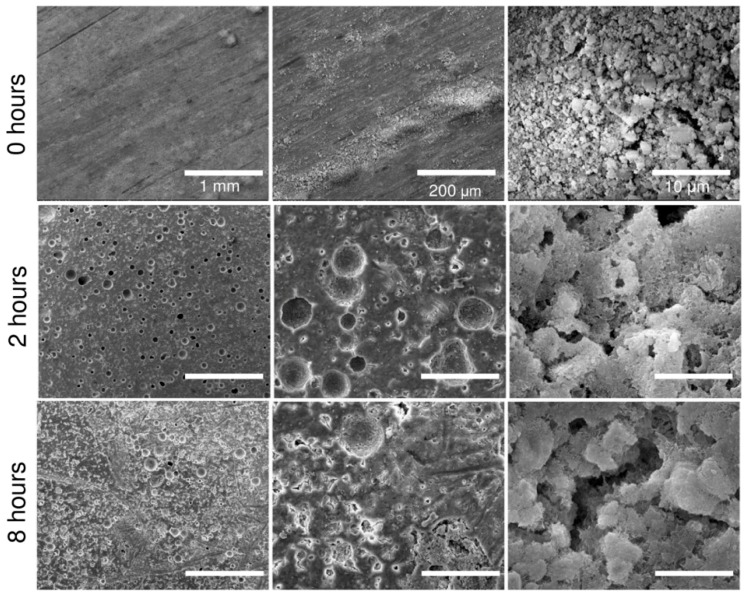
SEM images of the microstructure of the CPCs with the 20 *w*/*v*% copolymer and an L/P ratio of 0.5 g·g^−1^ (set for 10 days at 37 °C) after 0, 2 and 8 h of accelerated degradation with a scale bar of 1 mm (left), 200 μm (centre) and 10 μm (right).

The compressive strength of the set CPC samples with the 20 *w*/*v*% copolymer and an L/P of 0.5 g·g^−1^ decreased with the progression of mass loss as assessed by means of the conducting of an accelerated degradation study (Figure 7b). The acidic solution employed in the experiment (pH of 2.0–2.2) simulated the micro-environment beneath adherent macrophages and osteoclasts which led to localized degradation in the form of spherical pores on the surface of the cement (Figure 8). The morphology of the pores was like that of resorption pits generated by osteoclasts [24,25,26]. A more detailed observation at higher magnification revealed the disappearance of the plate-like crystal microstructure as a consequence of the dissolution of the CDHA crystals, which was corroborated by the increase in the calcium ion concentration in the immersion medium (Figure 7c). The rate of calcium ion release followed the first order kinetic release under the current accelerated conditions.

### 2.8. Cytocompatibility Testing

**Figure 9:**
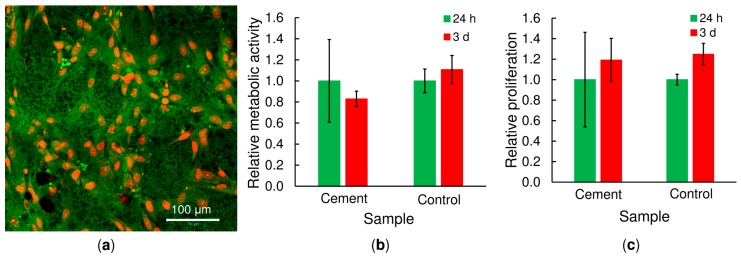
Cytocompatibility test on hMSCs; the cell morphology after 3 days of seeding on copolymer/*α*-TCP disks (20 *w*/*v*% copolymer, L/P of 0.5 g·g^−1^; setting for 10 days), cells seeded on scaffolds were visualized using confocal microscopy (DiOC6(3) – green color; propidium iodide – red color), scale bar = 100 μm (**a**). Cell metabolic activity was determined using MTS test (**b**); cell proliferation was determined in terms of the quantification of the amount of DNA at 24 hours and 3 days (**c**). Cells seeded on tissue culture plastic were used as a control and the data in b and c were normalized to the values obtained after 24 h of cell culture.

A preliminary cytocompatibility test was performed via the culturing human mesenchymal stem cells (hMSC) in direct contact with the copolymer/*α*-TCP cement (20 *w*/*v*% copolymer, L/P of 0.5 g·g^−1^, 10 days of setting in water at 37 °C). The cells were attached to the CPC surface and exhibited the stretched well-elongated shape typical of cultured hMSCs (Figure 9a). Figure 9b shows how the metabolic activity of the MSCs evolved during the experiment. The metabolic activity was determined using MTS test and the data were normalized to the values obtained after 24 h of cell culture. No statistically significant differences were observed in cell metabolic activity between 1 and 3 days of cell culture and the cement and the control groups followed a similar trend. The number of cells attached to the surface of the cement (cell proliferation) was determined by means of DNA quantification and the data were normalized to the values obtained after 24 h of cell culture (Figure 9c). Despite no statistically significant differences were observed for bone cement (*p* > 0.05), the results show an increase in the number of cells between 1 and 3 days of culture (*p* = 0.03 for control group). The results of cell metabolic activity and proliferation demonstrate the non-toxicity of the copolymer-based calcium phosphate cement. The low cellular metabolic activity was attributed to the complex microstructure of the cement together with ionic interactions with the cell culture medium, as reported in previous studies with similar materials [27].

## 3. Discussion

In general, the characterization of the visco-elastic properties of CPCs is a complex task due to the influence of the setting reaction [28]. In addition, no method is available for the determination of the setting of the cement in correlation to the evolution of the hydrolysis reaction (Equation (1)). Commonly, the setting time is determined by means of a mechanical method based on the indentation of one or two needles on the surface of the cement paste. Although these methods allow for a comparison of differing cement formulations, i.e., once they achieve certain strength, they do not directly correlate with the advancement of the hydrolysis reaction. Other methods that allow for the determination of the crystalline composition, such XRD, are not appropriate with respect to follow the initial setting of the cement since at very short time points, the changes in the crystalline composition are below the XRD detection limit. Rheological measurement provides the relevant information on the visco-elasticity of the pastes, as well as the setting process that reflects the evaluation of the internal (polymer or ion concentration, particle size, distribution, shape) and external (temperature) conditions during the self-hardening process [29]. It should be borne in mind that the setting reaction, shown in Equation (1), is based on the dissolution of α-TCP and the precipitation of CDHA [30]. Immediately following mixing, the dissolution of α-TCP regulates the process up to the commencement of the precipitation of the CDHA, from which point the dissolution of the α-TCP and the growth of new CDHA crystals progress in parallel. With concern to this mechanism, it is difficult to define when exactly the cement sets. Moreover, the dissolution rate of α-TCP depends on the environment [21]. Consequently, this study considered the use of ultrapure water only, i.e., without the presence of other inorganic additives, in order to gain a better understanding of the setting mechanism.

In general, setting is understood as the loss of cement paste workability, while hardening is defined as the increase in the strength of a solid cement block until a total chemical reaction is achieved. The question then is how to correlate workability with the chemistry. The microstructural analysis determined that the first visible CDHA crystals appeared after 6 h of reaction on the surface of the α-TCP particles. Nonetheless, CDHA diffraction peaks were not observed until after 12 h of reaction. Nevertheless, XRD provided information on the dissolution of the α-TCP particles in that the increase in the intensity of the diffraction peaks of the α-TCP could be explained by the dissolution of the most reactive domains of the α-TCP particles. After 6 h of reaction this trend was reversed with the occurrence of a decrease in the intensity of the diffraction peaks of the α-TCP that correlated with the time at which the first CDHA crystals were observed within the microstructure of the cement, thus possibly representing nucleation and the commencement of the growth of the CDHA crystals. The nucleation of the CDHA crystals after 6 h of reaction was further supported by the variation in the pH in the water in which the cement set. The pH increased up to a maximum value after 6 h of reaction due to the release of ions from the cement particles (Figure 4). Subsequently, and in correlation with the nucleation of the CDHA crystals, the pH decreased, since the nucleation and growth of the CDHA required the consumption of calcium, phosphate and hydroxyl ions. The pore size distribution (Figure 6b) also reflected the changes in the microstructure during the α-TCP reaction. Firstly, the pore size between the α-TCP particles increased due to the dissolution thereof; however, the volume of the pores decreased (thinner peak) due to the growth of the CDHA crystals. Finally, the spaces between the particles were completely filled by the CDHA crystals, resulting exclusively in pores of less than 1 µm in size.

**Figure 10:**
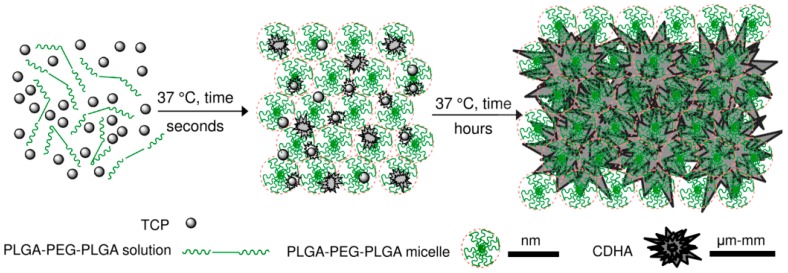
Scheme represents copolymer and α-TCP mixing followed by fast micellization of copolymer at 37 °C accompanied by both α-TCP dissolution and CDHA precipitation. With prolong time in the range of days, the CDHA crystals grow while packing together and increasing their size.

Previous analysis demonstrated that the nucleation of CDHA commenced after 6 h of the reaction between the copolymer water solution and the α-TCP; however, the means by which the workability of the cement changed during this time was addressed solely by means of time-sweep curves of the cement, which demonstrated a sharp increase in the storage modulus thus indicating a change in the microstructure and nucleation development of the CDHA. Prior to the sharp increase in the storage modulus (within the lag phase), the cement pastes exhibited, in general, a constant storage modulus, the magnitude of which depended on parameters, including the L/P ratio, copolymer content and temperature. It is a general concept that temperature significantly affects the storage modulus of thermo-sensitive copolymers; such copolymers convert from the liquid to the gel state with an increase in temperature via micelle-packing and gel formation [18]. Moreover, the entire self-setting process is predominantly accelerated by temperature [20]. However, as the scheme in Figure 10 shows, while the gelation of copolymers is rapid (a few seconds), the setting of α-TCP takes a number of hours to several days. Therefore, as shown in Figure 2, the stiffness of cement pastes prior to CDHA nucleation is controlled by the degree of gelation of the copolymer which, in turn, directly depends on the concentration of the copolymer and the temperature.

The effect of temperature on the setting reaction was observed as a shorter lag phase or, in other words, the more rapid nucleation of the CDHA due to temperature promoting the dissolution of α-TCP. The addition of the copolymer significantly reduces the storage modulus of the cement in the lag phase, since the copolymer acts as a surfactant that decreases the interparticle forces within the cement. In contrast, when mixed with water, the α-TCP particles in the cement are subjected to attractive interaction forces which act to form a stiff material. Such pastes with high solid contents are very difficult to inject due to phase-separation [31,32]. It should be noted that when mixed with the copolymer, the nucleation of the CDHA was clearly observed as a sharp increase in the storage modulus, whereas when mixed with water no well-defined change in the modulus was observed. It should be possible via the conducting of a longer rheological experiment to observe the increase in the storage modulus of cements mixed with water, in a similar way to the increase in compressive strength. Therefore, in contrast to standard methods that determine setting times via indentation, time-sweep curves are able to discern between “false” setting due to the higher compaction of the cement as caused by a decrease in the L/P ratio and “real” setting caused by the attainment of local conditions for CDHA nucleation in a shorter time. In addition, since the setting time determined by needle indentation may differ from that determined by means of time-sweep curves, a new criterion other than the 15 min of final setting time proposed by Driessens et al. [33] will be required with respect to the selection of cements to be employed in clinical applications.

In contrast to the time-sweep curves obtained at a very low frequency and strain level, steady rheological analysis and viscosity curves obtained by the gradual increase of shear strain provide information on the workability, injectability and washout resistance of the cements. The yield stress observed in the stress-strain curves reflects the yield force required to start the injection of the cement paste [19]. The yield shear stress for the α-TCP paste with water was 3 times higher than that of the α-TCP paste with the copolymer. The difference was due to the surfactant nature of the copolymer that acts to reduce the attractive colloidal forces between the α-TCP particles. Congruently, it was very difficult to inject the α-TCP paste with water. In contrast, the lower yield stress of the α-TCP paste with the copolymer allowed for the easy injection of the paste through a 20 G needle without the occurrence of phase separation signals.

While yield stress defines the ease with which the cement can be injected, the trend in the reduction of shear stress following maximum stress and the recovery of viscosity once the shear strain is reduced provides information on phase separation during injection and paste cohesion when immersed in liquids. On the one hand, the pastes that retained a high level of shear stress when the shear strain increased did not exhibit phase separation during either the rheological or injection tests, thus allowing for minimally invasive implantation. However, on the other, those pastes that underwent a drastic reduction in shear stress following yield stress exhibited a tendency towards phase separation. Figure 3c,e clearly show both how the α-TCP paste prepared with water separated following rheological testing and how this is in concordance with disintegration in contact with water. In contrast, the α-TCP paste prepared with the copolymer remained homogeneous following rheological testing (Figure 3d), which is in agreement with both ease of injection, as can be seen by the formation of a filament, and cohesion, since the filament preserves its shape in contact with water (Figure 3f). Conversely, the high shear stress following yield together with the more rapid recovery of the viscous state prevented the occurrence of the disintegration of the paste in contact with liquids, thus providing anti-washout resistance. In other words, after placing the sample in position, the attraction interactions within the paste reform once the paste is at rest. The faster the re-formation of the attractive interactions, the shorter the cohesion time of the cement (minimum time required from mixing to observing anti-washout resistance). Note that at this time scale, the increase in attractive interactions is almost independent of the progress of the setting reaction and mainly depends on the pseudoplastic nature of the copolymer. However, a fast setting reaction contributes towards increasing the cohesion of the cement over a shorter time. In fact, Figure 3 clearly shows how the thermo-sensitive copolymer changes the behaviour of the cement paste from extremely thixotropic to increasingly pseudoplastic. This phenomenon is thought to be due to the intrinsic pseudoplastic behaviour of the copolymer promoted by gelation upon an increase in temperature. Thus, in addition to quantitatively assessing the setting behaviour of the α-TCP, rheological testing also provides a valuable tool for the prediction of the cohesion of CPC pastes.

Due to the force measurement limitations inherent to rheometers, it is necessary to study the hardening of the copolymer/*α*-TCP by means of a different method, commonly the compression test. Results have shown that compressive strength follows the same trend as the percentage of reaction, since the strength of the solid cement depends on the interlocking of the CDHA crystals, which increases along with CDHA growth. The accelerated degradation test was inspired by the acidic milieu attaining values of below pH 3 generated by osteoclasts in the bone resorption process and resulting in the dissolution of underlying minerals [25]. Even though this study does not cover the complexity of the degradation that occurs in the body [24], it does demonstrate the mass loss of the cement and the release of calcium ions over the exposure time. Moreover, the degradation of the cement generates pores on the surface with size and morphology similar to the reabsorption pits produced by osteoclasts, thus leading to a reduction in the compressive strength of the cement. A low pH is also able to catalyse the breakage of the ester linkage of the copolymer backbone and lead to higher polymer hydrolytic degradation [34,35]. Glycolic and lactic acid monomers produced upon PLGA degradation are further able to catalyse the dissolution of the cement [36], which may present an advantage for this new cement formulation in that it is potentially able to overcome the long resorption times observed in vivo with respect to other cement formulations [37]. A further approach to increasing the resorption rate of the cement consists of the incorporation of pores larger than 100 μm, which will allow for bone ingrowth and cement resorption [38]. Interestingly, the use of the copolymer did not exhibit cytotoxicity in contact with hMSC. Indeed, the cells exhibited a typical hMSC morphology, indicating that these scaffolds exerted no notable adverse effects on cell viability in this short-term in vitro study. Since cells may detach following prolonged static culturing due to the low calcium concentration, it is planned that soon, we will employ a hydrodynamic culturing system [39] in order to evaluate cytocompatibility over an extended culturing period. Moreover, we propose to compile an assessment of the novel copolymer/*α*-TCP cement in terms of normal immunological response using a large animal model (a White pig) in order to confirm its regenerative performance in vivo. The afore-mentioned properties indicate that this material presents a very promising system for use in mini-invasive surgery especially with concern to the delivery of thin-needle injection applications into bone defects and the fixation of comminuted bone fractures.

## 4. Materials and Methods 

### 4.1. Chemicals 

D,L-lactide (LA, ≥ 99.9%) and glycolide (GA, ≥ 99.9%) were supplied by Polysciences (Warrington, PA, USA). Poly(ethylene glycol), Stannous 2-ethylhexanoate (95%), calcium carbonate (CaCO_3_, ≥ 99.0%) and calcium hydrogen phosphate (CaHPO_4_, ≥ 98.0%) were purchased from Sigma-Aldrich (St. Louis, MO, USA). The ultrapure water (Type 1 according to ISO 3696) was prepared using the Millipore purification system (Milli-Q Academic, Millipore, Molsheim, France).

### 4.2. Synthesis of PLGA–PEG–PLGA Triblock Copolymers (Liquid Component of the CPC)

The PLGA–PEG–PLGA triblock copolymer with a theoretical molecular weight of 5250 g·mol^−1^, an LA/GA molar ratio of 3.0 and a PLGA/PEG weight ratio of 2.5 was synthetized in a nitrogen atmosphere via the ring opening polymerization method (Supplementary Figure S5) in a bulk according to Michlovská et al. [18]. Briefly, a one-pot reaction of calculated D,L-lactide, glycolide, poly(ethylene glycol) and stannous 2-ethylhexanoate proceeded in the nitrogen atmosphere in a high-vacuum all-glass pipe line at 130 °C for 3 h. Any unreacted chemicals were removed by means of rinsing the product three times with 80 °C ultrapure water. The resulting freeze-dried copolymer was dissolved in ultrapure water at 4 °C thus providing 10 *w*/*v*% and 20 *w*/*v*% solutions.

### 4.3. Synthesis of α-Tricalcium Phosphate (Powder Component of the CPC)

A well-established method was used to synthetize the *α*-TCP at 1400 °C via a solid-state reaction from a 2:1 molar mixture of CaCO_3_ and anhydrous CaHPO_4_ followed by air quenching so as to prevent the formation of the *β*-TCP polymorph [19]. The block obtained was dry milled for 15 min at 450 rpm in a planetary mill (Pulverisette 6, Fritsch Gmbh, Weimar, Germany) using an agate jar and balls (10 balls of 30 mm in diameter). The resulting powder was then sieved over 30 min using a 40 µm sieve (Filtra, Barcelona, Spain), introducing 145 g of powder together with 200 g of Zirconia balls (1.5 mm diameter; Tosoh, Tokyo, Japan).

### 4.4. Molecular Weight Analysis

The molecular weight, LA/GA molar ratio and PLGA/PEG weight ratio of the copolymer were determined by means of proton nuclear magnetic resonance ^1^H NMR spectroscopy on a Bruker (Billerica, MA, USA) ADVANCE III HD 700 MHz instrument in deuterated chloroform (CDCl_3_) solvent at 25 °C (Supplementary Figure S1). The number-average molecular weight (*M_n_*) and polydispersity index (*M_w_*/*M_n_*) of the PLGA–PEG–PLGA copolymer were determined via gel permeation chromatography with the multi-angle light scattering method (GPC-MALS) (Supplementary Figure S2). The instrumental setup included an Agilent HPLC 1100 Series instrument with a degasser, pump, autosampler, set of two PLgel 5 μm Mixed-C 300 × 7.5 mm columns (Agilent, Santa Clara, CA, USA) thermostated to 25 °C and a UV-VIS diode array detector connected to a DAWN HELEOS II multi-angle laser light scattering detector, a ViscoStar-II differential viscometer and Optilab T-rEX refractive index detectors (Wyatt Technology, Dernbach, Germany). Tetrahydrofuran was used as the mobile phase at a flow rate of 1 mL·min^−1^. 

### 4.5. Particle Size Analysis

The particle size distribution of the α-TCP powder was determined using a laser diffraction particle size analyser (LA-950, Horiba, Kyoto, Japan), and the specific surface area (SSA) of the powder was measured by means of nitrogen adsorption according to the 5-point Brunauer, Emmett and Teller (BET) method (Autosorb iQ-XR, Quantachrome Instruments, Boynton Beach, FL, USA).

### 4.6. Rheological Analysis

The rheological properties and tests (including the sol-gel transition, thixotropy, viscosity and dynamic time sweep tests) were conducted in steady mode using a controlled-stress rheometer (AR-G2, TA Instruments, New Castle, DE, USA). 

#### 4.6.1. Copolymer Thermo-Sensitivity Measurement

Cone-plate geometry with a diameter of 40 mm and a 2° angle was used for the temperature dependence measurement of the PLGA–PEG–PLGA triblock copolymer aqueous solution, which was transferred to the Peltier by means of a spatula. The working position was set with a geometry gap of 60 µm. A solvent trap filled with water was employed before the recording of the measurements so as to prevent the evaporation of the sample solvent. Experimental conditions were applied at a constant frequency of 1 rad s^−1^ and 1% strain. The temperature ramp was set up at from 15 °C to 60 °C and a rate of 0.5 °C per minute.

#### 4.6.2. Copolymer/*α*-TCP Setting Kinetics

The time sweep test was conducted with plate-plate geometry (diameter 20 mm), at a frequency of 1 Hz, a points delay time of 10 s, a deformation of 0.01%; the measurement time was 5 h at a temperature of 23 °C for the first set of samples and 37 °C for the second set. The first set of samples included three types of paste with L/P ratios of 0.35; 0.5; 0.65 g·g^−1^ concerning which a liquid thermo-sensitive copolymer (with a concentration of 10 *w*/*v*% or 20 *w*/*v*%) or water (0 *w*/*v*%) were used as the liquid phase. The second set of samples at both 23 °C and 37 °C included pastes with an L/P ratio of 0.5 g·g^−1^ and differing in the amount of the polymer. The rheological measurements were taken 3 min following the mixing of the two components.

#### 4.6.3. Copolymer/*α*-TCP Thixotropy and Viscosity

Parallel plate geometry with a diameter of 20 mm was used for measurement purposes with respect to the steady state rheological analysis. Samples of the prepared CPCs (0.16 mL) were transferred to the Peltier by means of a syringe. The AR-G2 rheometer (TA Instruments, New Castle, DE, USA) was set at the working position with a geometry gap of 500 µm. A solvent trap filled with water was employed so as to prevent solvent evaporation during time-sweep testing. The experimental conditions at 23 °C commenced at a shear rate of 1 s^−1^ which was maintained for 20 s followed by a rate increase to 100 1 s^−1^ which was also maintained at 20 s and a subsequent return to a shear rate of 1 s^−1^ according to the following scheme: $1s^{-1}\overset{20s}{\to }100s^{-1}\overset{20s}{\to }1s^{-1}$. Each sample was measured at least three times.

### 4.7. Microstructure Observation

After 1 min of the mixing of *α*-TCP powder and a 20 *w*/*v*% polymer solution as the liquid phase, the cement paste with an L/P ratio of 0.5 g·g^−1^ was placed in cylindrical rubber moulds with an internal diameter of 6 mm and a height of 9 mm. The samples were then immersed in ultrapure water at 37 °C. At selected time-points (3, 6, 12 and 24 h, 3, 7, 14 and 28 days) one of the samples was extracted, rinsed with distilled water and quenched with liquid nitrogen so as to prevent a hydrolysis reaction and, finally, freeze-dried (EPSILON 2D-10, Martin Christ, Osterode am Harz, Germany). The morphology of the initial α-TCP powder and set cements was investigated employing a scanning electron microscope (SEM, Tescan Mira3, Brno, Czech Republic). All the observations were made in the secondary electron emission mode with a 10 kV acceleration voltage, beam intensity of 10 and a working distance of 15 mm. In order to ensure good resolution, the samples were coated with a 20 nm layer of gold/palladium. 

### 4.8. Kinetics of the α-TCP Transformation to CDHA

The kinetics of the transformation from the original α-TCP to calcium-deficient hydroxyapatite (CDHA) of the CPC with the 20 *w*/*v*% polymer solution and L/P ratio of 0.5 g·g^−1^ was studied by means of Wide-Angle X-ray Scattering Analysis (WAXS, Rigaku Miniflex 6000, Tokyo, Japan). Following SEM observation, the remaining set cement samples were milled and analysed in Bragg-Brentano geometry, with an accelerating voltage of 40 kV and 15 mA, Cu-Kα radiation, with a D/teX detector, filter K(β) and a step width of 0.02° in a scanning range of 2° to 60° at laboratory temperature (21 ± 0.5 °C). The diffraction patterns of the analysed samples were collected and evaluated employing PDXL software.

### 4.9. Porosity Determination

Changes in the pore size distribution in the cement during setting were investigated by means of mercury intrusion porosimetry (PoreMaster 60, Quantachrome Instruments, Boynton Beach, FL, USA). The cement samples set over 10, 24 and 35 h, 3, 7 and 14 days by means of immersion in ultrapure water at 37 °C and, at the specified time-points, were withdrawn and freeze-dried (Martin Christ lyophilizator EPSILON 2D-10, Osterode am Harz, Germany). 

### 4.10. Compression Testing

Compression testing was performed on the cement cylinders prepared as described in the “Microstructure observation” section using mechanical testing equipment (Zwick Roell 022, Ulm, Germany) with a load-cell of 1 kN, a crosshead speed of 1 mm min^−1^ and a pre-load of 10 g. The compression testing of the samples at different reaction times and the samples immersed in the acidic solution for 4 and 8 h (see the “Accelerated degradation study”) was performed according to the ISO 13,314 standard [40]. The ultimate compressive strength was calculated following the measurement of the ultimate compressive force. The mechanical data was collected in testXpert II software (Zwick Roell, Ulm, Germany) and each set consisted of 5 samples. 

### 4.11. Accelerated Degradation Study

Accelerated degradation testing was conducted according to Escudero et al. [24]. Cylindrical samples (*n* = 5 for each time-point) with dimensions of 9 mm in diameter and 2 mm in height made from *α*-TCP mixed with the 20 *w*/*v*% copolymer (L/P ratio of 0.5 g·g^−1^ set for 14 days) were dried at 120 °C overnight until a constant weight was achieved. Cylindrical samples with an acidic solution consisting of 0.01 M hydrochloric acid (HCl, Penta, Praha, Czech Republic) and 0.14 M sodium chloride (NaCl, Penta, Praha, Czech Republic) (pH = 2.2 at 37 °C) were placed in polypropylene tubes for 8 h. The solution was changed every hour and the pH values were checked at each time-point in the collected supernatants using an IKATRON THETA 90 electrode (IKA Werke, Staufen im Breisgau, Germany). The ratio of the solution volume and the surface area of the samples was kept constant (100 mL to 185 mm^2^) for all the tests so as to maintain a constant pH during degradation testing.

#### Mass Loss and Calcium Ion Release

Following degradation, the samples were withdrawn from the acidic solution at specified times, washed three times in deionized water and dried at 120 °C until constant weight was achieved. The mass loss percentage was calculated according to the following Equation (2):

(2) $$mass\;loss=\;\frac{w_{0}-w_{t}}{w_{0}}100\;\left[\%\right],$$

where *w*_0_ is the initial dried weight of the sample and *w_t_* is the dried weight of the sample following degradation. The calcium concentration of the solution was measured at each time-point using an HI4104 calcium ion selective electrode (HANNA Instruments, Woonsocket, RI, USA).

### 4.12. Cytocompatibility Testing

The cytocompatibility of the bone cements after 10 days of setting was tested using human mesenchymal stem cells (hMSCs; ScienCell, Carlsbad, CA, USA). Copolymer/*α*-TCP samples (L/P ratio of 0.5, 20 *w*/*v*% of copolymer) with a diameter of 6 mm were sterilized using ethylene oxide and seeded with hMSCs at a density of 2 × 10^4^ per sample. The cells were cultured in αMEM supplemented with 10% foetal bovine serum (FBS) and 1% antibiotics (Penicillin/Streptomycin). Cells seeded on tissue culture plastic were used as a control group.

#### 4.12.1. DNA Quantification

On days 1 and 3, the amount of cells on the scaffolds (cell proliferation) was determined by means of dsDNA quantification (Quant-iT™ dsDNA Assay Kit; Life Technologies, Carlsbad, CA, USA). The polymer/*α*-TCP samples seeded with cells were placed in 300 μL of cell lysis solution (0.2% *v*/*v* Triton X-100, 10 mM Tris (pH 7.0), and 1 mM EDTA) and processed through 3 freeze/thaw cycles (vortexed between each cycle). The samples were further processed according to the manufacturer’s instructions and measured using a multiplate fluorescence reader (Synergy HT, Winooski, VT, USA; λex = 485 nm, λem = 525 nm). The averaged dsDNA quantification values were determined after 24 and 72 h with respect to at least 5 independently prepared samples. The data were normalized to the values obtained after 24 h of cell culture.

#### 4.12.2. Cell Distribution

Confocal microscopy was used for the visualisation of the distribution of cells on the cements. The copolymer/*α*-TCP samples were fixed with frozen methyl alcohol (−20 °C) for 10 min and subsequently incubated with 3,3′-dihexyloxacarbocyanine iodide (DiOC6(3) (D273, Invitrogen, Molecular Probes, Eugene, OR, USA, 1 μg·mL^−1^ in phosphate buffered saline (PBS); pH 7.4) for 45 min at room temperature so as to visualize the cell membranes (in green). The samples were then incubated with propidium iodide (PI; 5 μg·mL^−1^ in PBS; Sigma-Aldrich, St. Louis, MO, USA) for 10 min so as to visualize the cell nuclei (in red). The samples were scanned using a ZEISS LSM 5 DUO confocal microscope, Oberkochen, Germany (PI: λex = 561 nm, λem = 630 − 700 nm; DiOC6 (3): λex = 488 nm, λem = 505 − 550 nm).

#### 4.12.3. MTS Test

The MTS assay (CellTiter96^®^ AQueous One Solution Cell Proliferation Assay, Promega, WI, USA) was used 24 and 70 h after seeding to determine the metabolic activity of the cells on the bone cements. Briefly, the scaffolds were transferred into new wells to prevent the cells adhered to the tissue culture plastic to misrepresent the measured data. Subsequently, 100 μL of fresh media and 20 μL of the MTS substrate were added to each well. After 2 h of incubation at 37 °C, 100 μL of the cultured solution was transferred to a new clean well. The absorbance of the media was detected at 490 nm using a multi-mode microplate reader Synergy HT (BioTek Instruments, Winooski, VT, USA). The background absorbance (690 nm) and the absorbance of the medium without cells were subtracted from the measured absorbance. The data were normalized to the values obtained after 24 h of cell culture.

### 4.13. Statistical Analysis

The mean values were calculated for the number of readings (*n*) from each experiment; the error bars refer to the standard deviation (SD). The cytocompatibility results were evaluated statistically using the One Way Analysis of Variance method and the Student-Newman-Keuls test (SigmaStat 12.0, Systat Software, San Jose, CA, USA). Statistical significance was accepted when *p* ≤ 0.05.

## 5. Conclusions

Rheology testing provides a valuable tool for the quantitative determination of the setting time, injectability and anti-washout resistance of α-TCP cements. The setting time corresponds to the lag phase in the time-sweep curves and the increase in the storage modulus correlates well with the commencement of the nucleation of CDHA crystals. Although α-TCP cements are thixotropic fluids, their visco-elastic properties can be controlled via the addition of a thermo-sensitive PLGA–PEG–PLGA copolymer. As has been demonstrated, the use of this copolymer is interesting, since it provides for the enhanced pseudoplastic behaviour of the α-TCP cement and increases the viscosity thereof due to gelation. The cements maintain high shear stress with an increase in the shear rate, thus preventing phase separation during injection. Moreover, copolymer/α-TCP cements quickly recover their viscous state upon the disappearance of shear, which prevents the washout of the cements when immersed in liquids. As a consequence of the afore-mentioned properties and the positive preliminary results obtained from the cytocompatibility tests performed on hMSC, it can be concluded that the novel injectable composite copolymer/α-TCP bone cement studied herein is suitable for mini-invasive surgery application.

## Figures and Tables

**Table 1:** The effect of PLGA–PEG–PLGA copolymer concentration (c = 0; 10 and 20 *w*/*v*%) and the L/P ratio (0.35; 0.5 and 0.65 g·g^−1^) at 23 °C on the pH, complex viscosity (η*) and injectable/cohesion properties of the copolymer/α-TCP cements 3 min following mixing (in the lag phase).

L/P (g·g^−1^)	0.35			0.5			0.65		
c (*w*/*v*%)	pH	η* (Pa·s)	Injectable/Cohesion	pH	η* (Pa·s)	Injectable/Cohesion	pH	η* (Pa·s)	Injectable/Cohesion
0	8.4	206,000	No/No	8.5	1436	Poorly/No	8.6	345	Yes/No
10	7.4	3.2	Yes/Yes	7.3	2.9	Yes/Yes	7.2	2.5	Yes/Yes
20	7.1	43.4	Yes/Yes	7.0	12.1	Yes/Yes	7.0	4.8	Yes/Yes

## Supplementary Materials

Supplementary Materials can be found at http://www.mdpi.com/1422-0067/20/2/391/s1. Figure S1: ^1^H NMR spectrum of PLGA–PEG–PLGA copolymer. Molecular weight and molar composition of copolymer were determined from integrals of characteristic proton intensities of lactic acid (O(CH_3_)CHO) in a range between δ = 5.1–5.35 ppm (multiplet, 1H) (a) and (OC(CH_3_)CHO) protons at δ = 1.5–1.75 ppm (multiplet, 3H) (e), glycolic acid (OCH_2_O) at δ = 4.6–4.9 ppm (multiplet, 2H) (b), PEG (OCH_2_CH_2_O) at δ = 3.55–3.75 ppm (multiplet, 3H) (d). The real molecular weight of the copolymer is 5210 g·mol^−1^, PLGA/PEG weight ratio is 2.47 and LA/GA molar ratio is 2.96. Figure S2: Gel permeation chromatography with the multi-angle light scattering (GPC-MALS) of PLGA-PEG-PLGA copolymer. The measured number-average molecular weight (*M_n_*) and polydispersity index (*M_w_*/*M_n_*) were 5300 g·mol^−1^ and 1.23, respectively. Figure S3: Scanning electron micrograph (a) and particle size distribution (b) for α-TCP powder component of the CPCs. Figure S4: Time-sweep curves of α-TCP cement pastes with different L/P ratio (0.35; 0.5; 0.65 g·g^−1^) and 20 *w*/*v*% polymer solution at 23 °C. Figure S5: Synthesis of the PLGA–PEG–PLGA triblock copolymer via ring-opening polymerization technique.

## References

[1] Bohner M., Gbureck U., Barralet J. (2005). Technological issues for the development of more efficient calcium phosphate bone cements: A critical assessment. Biomaterials.

[2] Ginebra M., Canal C., Espanol M., Pastorino D., Montufar E. (2012). Calcium phosphate cements as drug delivery materials. Adv. Drug Deliv. Rev..

[3] O’Hara R., Buchanan F., Dunne N. (2014). Injectable calcium phosphate cements for spinal bone repair. Biomater. Bone Regen..

[4] O’Hara R., Orr J., Buchanan F., Wilcox R., Barton D., Dunne N. (2012). Development of a bovine collagen–apatitic calcium phosphate cement for potential fracture treatment through vertebroplasty. Acta Biomater..

[5] Perez R., Ginebra M. (2013). Injectable collagen/α-tricalcium phosphate cement: Collagen–mineral phase interactions and cell response. J. Mater. Sci. Mater. Med..

[6] Bigi A., Bracci B., Panzavolta S. (2004). Effect of added gelatin on the properties of calcium phosphate cement. Biomaterials.

[7] An J., Wolke J., Jansen J., Leeuwenburgh S. (2016). Influence of polymeric additives on the cohesion and mechanical properties of calcium phosphate cements. J. Mater. Sci. Mater. Med..

[8] Song H., Esfakur Rahman A., Lee B. (2009). Fabrication of calcium phosphate-calcium sulfate injectable bone substitute using chitosan and citric acid. J. Mater. Sci. Mater. Med..

[9] Wang X., Ye J., Wang H. (2006). Effects of additives on the rheological properties and injectability of a calcium phosphate bone substitute material. J. Biomed. Mater. Res. Part B.

[10] Shahbazi S., Moztarzadeh F., Sadeghi G., Jafari Y. (2016). In vitro study of a new biodegradable nanocomposite based on poly propylene fumarate as bone glue. Mater. Sci. Eng. C.

[11] Maazouz Y., Montufar E., Malbert J., Espanol M., Ginebra M. (2017). Self-hardening and thermoresponsive alpha tricalcium phosphate/pluronic pastes. Acta Biomater..

[12] Huh H., Zhao L., Kim S. (2015). Biomineralized biomimetic organic/inorganic hybrid hydrogels based on hyaluronic acid and poloxamer. Carbohydr. Polym..

[13] Qiao M., Chen D., Ma X., Liu Y. (2005). Injectable biodegradable temperature-responsive PLGA–PEG–PLGA copolymers: Synthesis and effect of copolymer composition on the drug release from the copolymer-based hydrogels. Int. J. Pharm..

[14] Rizzarelli P., Carroccio S. (2014). Modern mass spectrometry in the characterization and degradation of biodegradable polymers. Anal. Chim. Acta.

[15] Yu L., Chang G., Zhang H., Ding J. (2007). Temperature-induced spontaneous sol-gel transitions of poly(d,l-lactic acid-co-glycolic acid)-b-poly(ethylene glycol)-b-poly(d,l-lactic acid-co-glycolic acid) triblock copolymers and their end-capped derivatives in water. J. Polym. Sci. Part A Polym. Chem..

[16] Michlovská L., Vojtová L., Humpa O., Kučerík J., Žídek J., Jančář J. (2016). Hydrolytic stability of end-linked hydrogels from PLGA–PEG–PLGA macromonomers terminated by α,ω-itaconyl groups. RSC Adv..

[17] Chamradová I., Vojtová L., Částková K., Diviš P., Peterek M., Jančář J. (2017). The effect of hydroxyapatite particle size on viscoelastic properties and calcium release from a thermosensitive triblock copolymer. Colloid Polym. Sci..

[18] Michlovská L., Vojtová L., Mravcová L., Hermanová S., Kučerík J., Jančář J. (2010). Functionalization Conditions of PLGA–PEG–PLGA Copolymer with Itaconic Anhydride. Macromol. Symp..

[19] Montufar E., Maazouz Y., Ginebra M. (2013). Relevance of the setting reaction to the injectability of tricalcium phosphate pastes. Acta Biomater..

[20] Durucan C., Brown P. (2000). α-Tricalcium phosphate hydrolysis to hydroxyapatite at and near physiological temperature. J. Mater. Sci. Mater. Med..

[21] Wei X., Ugurlu O., Akinc M. (2007). Hydrolysis of α-Tricalcium Phosphate in Simulated Body Fluid and Dehydration Behavior during the Drying Process. J. Am. Ceram. Soc..

[22] Espanol M., Perez R., Montufar E., Marichal C., Sacco A., Ginebra M. (2009). Intrinsic porosity of calcium phosphate cements and its significance for drug delivery and tissue engineering applications. Acta Biomater..

[23] Carte D., Hayes W. (1977). The compressive behavior of bone as a two-phase porous structure. J. Bone Jt. Surg. Ser. A.

[24] Diez-Escudero A., Espanol M., Beats S., Ginebra M.P. (2017). In vitro degradation of calcium phosphates: Effect of multiscale porosity, textural properties and composition. Acta Biomater..

[25] Silver I.A., Murrills R.J., Etherington D.J. (1988). Microelectrode studies on the acid microenvironment beneath adherent macrophages and osteoclasts. Exp. Cell Res..

[26] Sariibrahimoglu K., Leeuwenburgh S.C., Wolke J.G., Yubao L., Jansen J.A. (2012). Effect of calcium carbonate on hardening, physicochemical properties, and in vitro degradation of injectable calcium phosphate cements. J. Biomed. Mater. Res. Part A.

[27] Sadowska J.M., Guillem-Marti J., Montufar E.B., Espanol M., Ginebra M.P. (2017). Biomimetic versus Sintered Calcium Phosphates: The in vitro Behavior of Osteoblasts and Mesenchymal Stem Cells. Tissue Eng. Part A.

[28] Bohner M., Baroud G. (2005). Injectability of calcium phosphate pastes. Biomaterials.

[29] O’Neill R., McCarthy H., Montufar E., Ginebra M., Wilson D., Lennon A., Dunne N. (2017). Critical review: Injectability of calcium phosphate pastes and cements. Acta Biomater..

[30] Ginebra M., Fernández E., De Maeyer E., Verbeeck R., Boltong M., Ginebra J., Driessens F., Planell J. (2016). Setting Reaction and Hardening of an Apatitic Calcium Phosphate Cement. J. Dent. Res..

[31] Liu C., Shao H., Chen F., Zheng H. (2006). Rheological properties of concentrated aqueous injectable calcium phosphate cement slurry. Biomaterials.

[32] Sarda S., Fernández E., Llorens J., Martínez S., Nilsson M., Planell J. (2001). Rheological properties of an apatitic bone cement during initial setting. J. Mater. Sci. Mater. Med..

[33] Driessens F., Planell J., Boltong M., Khairoun I., Ginebra M. (2016). Osteotransductive bone cements. Proc. Inst. Mech. Eng. Part H.

[34] Zolnik B.S., Burgess D.J. (2007). Effect of acidic pH on PLGA microsphere degradation and release. J. Control. Release.

[35] Yoo J.Y., Kim J.M., Seo K.S., Jeong Y.K., Lee H.B., Khang G. (2005). Characterization of degradation behavior for PLGA in various pH condition by simple liquid chromatography method. Bio-Med. Mater. Eng..

[36] Smith B.T., Lu A., Watson E., Santoro M., Melchiorri A.J., Grosfeld E.C., van den Beucken J.J.J.P., Jansen J.A., Scott D.W., Fisher J.P. (2018). Incorporation of fast dissolving glucose porogens and poly(lactic-co-glycolic acid) microparticles within calcium phosphate cements for bone tissue regeneration. Acta Biomater..

[37] Frankenburg E.P., Goldstein S.A., Bauer T.W., Harris S.A., Poser R.D. (1998). Biomechanical and histological evaluation of a calcium phosphate cement. J. Bone Jt. Surg. Am..

[38] Kovtun A., Goeckelmann M., Niclas A., Montufar E., Ginebra M., Planell J., Santin M., Ignatius A. (2015). In vivo performance of novel soybean/gelatin-based bioactive and injectable hydroxyapatite foams. Acta Biomater..

[39] Schumacher M., Uhl F., Detsch R., Deisinger U., Ziegler G. (2010). Static and dynamic cultivation of bone marrow stromal cells on biphasic calcium phosphate scaffolds derived from an indirect rapid prototyping technique. J. Mater. Sci. Mater. Med..

[40] ISO13314:2011 (2011). Mechanical Testing of Metals—Ductility Testing–Compression Test for Porous and Cellular Metals.

