# Supplementary material for: The Effect of the Thermosensitive Biodegradable PLGA–PEG–PLGA Copolymer on the Rheological, Structural and Mechanical Properties of Thixotropic Self-Hardening Tricalcium Phosphate Cement
Source: Int J Mol Sci. 2019 Jan 17;20(2):391. doi: 10.3390/ijms20020391 (PMC6359562; doi:10.3390/ijms20020391)
Supplement: Supplementary file 1 [file ijms-20-00391-s001.pdf]

International Journal of *Molecular Sciences*

MDPI

Supplementary materials

The effect of the thermosensitive biodegradable PLGA-PEG-PLGA copolymer on the rheological, structural and mechanical properties of thixotropic self-hardening tricalcium phosphate cement

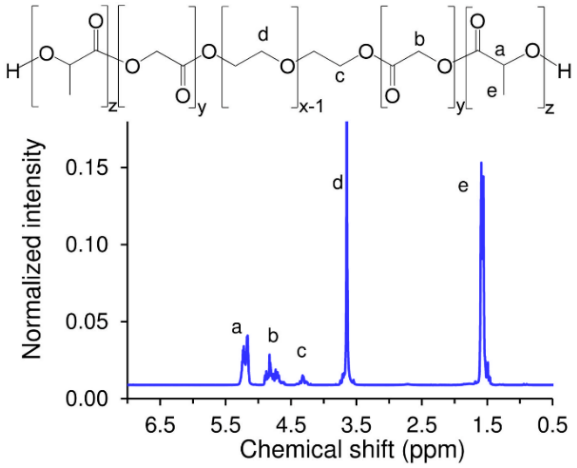

Figure S1. ^{1}H NMR spectrum of PLGA—PEG—PLGA copolymer. Molecular weight and molar composition of copolymer were determined from integrals of characteristic proton intensities of lactic acid (O(CH_3)CHO) in a range between δ = 5.1 - 5.35 ppm (multiplet, 1H) (a) and (OC(CH_3)CHO) protons at δ = 1.5 - 1.75 ppm (multiplet, 3H) (e), glycolic acid (OCH_2O) at δ = 4.6 - 4.9 ppm (multiplet, 2H) (b), PEG (OCH_2CH_2O) at δ = 3.55 - 3.75 ppm (multiplet, 3H) (d). The real molecular weight of the copolymer is 5 210 g.mol-1, PLGA/PEG weight ratio 2.47 and LA/GA molar ratio 2.96.

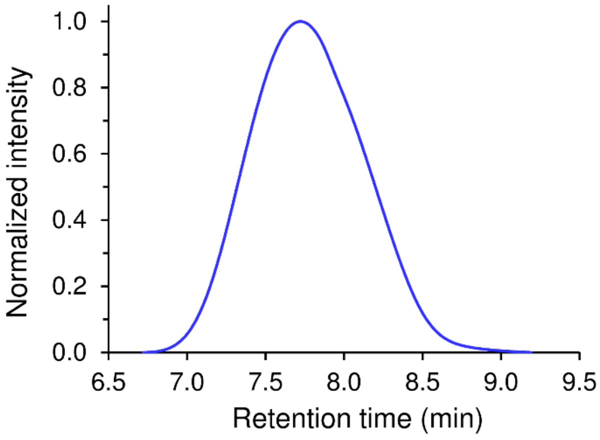

Figure S2. Gel permeation chromatography with the multi-angle light scattering (GPC-MALS) of PLGA-PEF-PLGA copolymer. The measured number-average molecular weight (Mn) and polydispersity index (Mw/Mn) were 5300 g.mol-1 and 1.23, respectively.

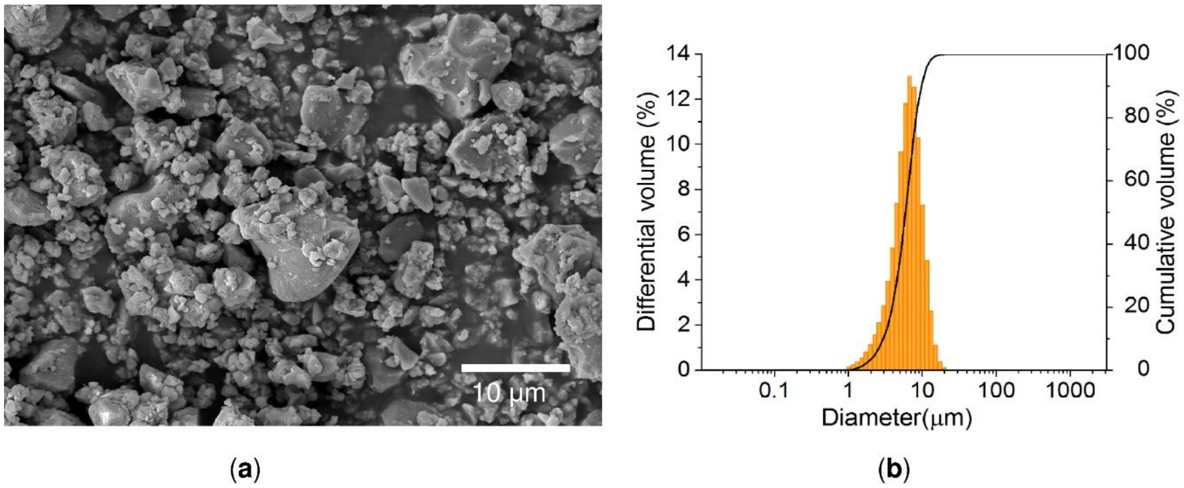

Figure S3. Scanning electron micrograph (a) and particle size distribution (b) for α-TCP powder component of the CPCs.

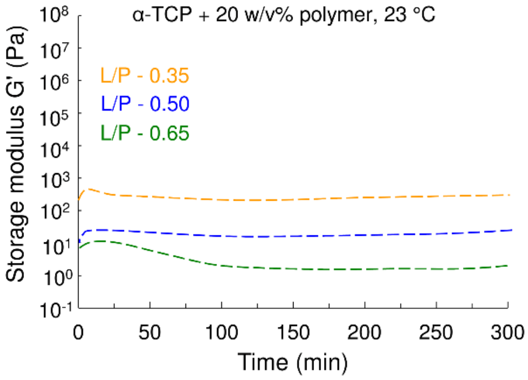

Figure S4. Time-sweep curves of α-TCP cement pastes with different L/P ratio (0.35; 0.5; 0.65 $g{\cdot}g^{-1}$) and 20 w/v % polymer solution at 23 °C.

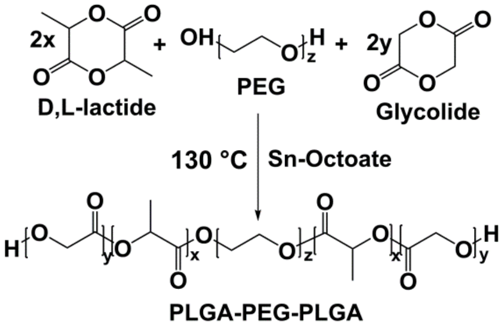

Figure S5. Synthesis of the PLGA—PEG—PLGA triblock copolymer via ring-opening polymerization technique.
